# Circulating FH Protects Kidneys From Tubular Injury During Systemic Hemolysis

**DOI:** 10.3389/fimmu.2020.01772

**Published:** 2020-08-07

**Authors:** Nicolas S. Merle, Juliette Leon, Victoria Poillerat, Anne Grunenwald, Idris Boudhabhay, Samantha Knockaert, Tania Robe-Rybkine, Carine Torset, Matthew C. Pickering, Sophie Chauvet, Veronique Fremeaux-Bacchi, Lubka T. Roumenina

**Affiliations:** ^1^Centre de Recherche des Cordeliers, INSERM, Sorbonne Université, Université de Paris, Paris, France; ^2^Centre for Complement and Inflammation Research, Imperial College London, London, United Kingdom; ^3^Assistance Publique – Hôpitaux de Paris, Service de Nephrologie, Hôpital Européen Georges Pompidou, Paris, France; ^4^Assistance Publique – Hôpitaux de Paris, Service d'Immunologie Biologique, Hôpital Européen Georges Pompidou, Paris, France

**Keywords:** complement – immunological term, complement factor H, hemolysis, kidney, acute tubular damage

## Abstract

Intravascular hemolysis of any cause can induce acute kidney injury (AKI). Hemolysis-derived product heme activates the innate immune complement system and contributes to renal damage. Therefore, we explored the role of the master complement regulator Factor H (FH) in the kidney's resistance to hemolysis-mediated AKI. Acute systemic hemolysis was induced in mice lacking liver expression of FH (hepatoFH^−/−^, ~20% residual FH) and in WT controls, by phenylhydrazine injection. The impaired complement regulation in hepatoFH^−/−^ mice resulted in a delayed but aggravated phenotype of hemolysis-related kidney injuries. Plasma urea as well as markers for tubular (NGAL, Kim-1) and vascular aggression peaked at day 1 in WT mice and normalized at day 2, while they increased more in hepatoFH^−/−^ compared to the WT and still persisted at day 4. These were accompanied by exacerbated tubular dilatation and the appearance of tubular casts in the kidneys of hemolytic hepatoFH^−/−^ mice. Complement activation in hemolytic mice occurred in the circulation and C3b/iC3b was deposited in glomeruli in both strains. Both genotypes presented with positive staining of FH in the glomeruli, but hepatoFH^−/−^ mice had reduced staining in the tubular compartment. Despite the clear phenotype of tubular injury, no complement activation was detected in the tubulointerstitium of the phenylhydrazin-injected mice irrespective of the genotype. Nevertheless, phenylhydrazin triggered overexpression of C5aR1 in tubules, predominantly in hepatoFH^−/−^ mice. Moreover, C5b-9 was deposited only in the glomeruli of the hemolytic hepatoFH^−/−^ mice. Therefore, we hypothesize that C5a, generated in the glomeruli, could be filtered into the tubulointerstitium to activate C5aR1 expressed by tubular cells injured by hemolysis-derived products and will aggravate the tissue injury. Plasma-derived FH is critical for the tubular protection, since pre-treatment of the hemolytic hepatoFH^−/−^ mice with purified FH attenuated the tubular injury. Worsening of acute tubular necrosis in the hepatoFH^−/−^ mice was trigger-dependent, as it was also observed in LPS-induced septic AKI model but not in chemotherapy-induced AKI upon cisplatin injection. In conclusion, plasma FH plays a key role in protecting the kidneys, especially the tubules, against hemolysis-mediated injury. Thus, FH-based molecules might be explored as promising therapeutic agents in a context of AKI.

## Introduction

Intravascular hemolysis is responsible for acute kidney injury (AKI), partly due to cellular damage induced by hemoglobin and its breakdown product heme (1). There are multiple nephrotoxic mechanisms of hemoglobin and heme, including direct toxicity, oxidative stress, vasoconstriction, NO scavenging and sterile inflammation.

Renal injury related to hemolysis has been described in genetic hemoglobinopathies (like sickle cell disease, SCD), in malaria or in transfusion of stored red blood cells, where hemolysis-derived products injure renal structures, particularly the proximal tubules (2, 3). Interestingly, these products and especially heme, have also been shown to activate the complement system (4).

Complement is a powerful plasmatic innate immune defense mechanism, which is tightly regulated to prevent tissue injury (5, 6). Recent studies have identified a key role of complement overactivation in tissue damage occurring in hemolytic diseases, including SCD (7–12). Notably, complement activation drives acute tubular injury, which was prevented in C3^−/−^ mice (5, 6, 8). One explanation for this phenomenon is that heme directly triggers the alternative complement pathway (AP) in the circulation. Moreover, heme also renders the endothelial cells (EC) susceptible to complement attack (7, 9, 13).

Despite these studies, the mechanisms of complement regulation in the context of hemolysis remain poorly understood. Factor H (FH) is a major plasma regulator of the complement system acting at level of C3 (5). By interacting with C3b, FH controls the complement cascade activation both in the bloodstream and on cell surfaces, preventing the terminal C5b-9 complex formation (14–18). Impaired FH binding to tubular cells in a mouse model of renal ischemia/reperfusion resulted in aggravation of the phenotype, emphasizing the protective effect of the circulating FH against acute tubular necrosis (19, 20). However, the role of FH in the context of hemolysis-related AKI remains unknown.

Moreover, systemic FH is mainly (up to 80%) produced by the liver (21). The kidney is considered as a major extrahepatic producer of complement proteins, but the capacity of its cells to produce and bind FH at resting state is debated.

Here we showed that systemic FH protected the kidneys from hemolysis-induced renal injury. We used a mouse model hepatoFH^−/−^ unable to produce hepatic FH to differentiate its role from local FH. These data identify the complement system as a key driver of tubular damage in hemolytic diseases and show the potential for administration of purified FH as a promising therapeutic strategy.

## Methods

### Reagents

Stock solution of 25 mg/ml Phenylhydrazin (PHZ) (Sigma-Aldrich) and of 250 μg/ml LPS (LPS-EB from *E. coli* O111:B4, InVivogen) were prepared in sterile PBS (Gibco) immediately before use.

### Animal Experimentation

Experimental protocols were approved by Charles Darwin ethical committee (Paris, France) and by the French Ministry of Agriculture (Paris, France) number APAFIS# 3764-201601121739330v3. All the experiments were conducted in accordance with their recommendations for care and use of laboratory animals. Two different strains, housed in specific germs-free conditions, were used: wild type C57BL/6 mice (WT) purchased from Charles River Laboratories (L'Arbresle, France) and hepatocyte-Cfh^−/−^ C57BL/6 mice (hepatoFH^−/−^) (21). HepatoFH^−/−^ mice have been generated by crossing alb-Cre (Jackson Laboratories, 003574) with FH–loxP mice (Cfh^loxP/loxP^).

### Mouse Treatment

Mice were injected with intraperitoneal (IP) injection of 100 μL PBS or PHZ (900 μmol/kg, corresponding to 0.125 mg/g body weight) and the mice were sacrificed at day 1, 2, or 4 (Figure S1A). Blood and urines were collected 3 days before the first injection (baseline). Blood, urines and organs, including kidneys, heart and liver, were recovered at sacrifice. Eight to 16-week-old mice were sex- and age-matched for each experiment. Mouse blood samples were collected into heparin–containing Eppendorf tubes. Organs were frozen in nitrogen liquid and stored at −80°C or fixed in 4% paraformaldehyde during 24 h for paraffin inclusion with HistoCore® (Leica Biosystem). Alternatively, PBS, Cisplatin (Tocris Bioscience 15 mg/kg) or LPS (InvivoGen, 2 mg/kg) were injected IP and mice were sacrificed at day 2. To test the efficacy of FH, mice were pretreated with 2 intravenous injections of 250 μL human FH 1 mg/mL (corresponding to 0.025 mg/g body weight), at 3 h and 1 h before IP injection of PBS or PHZ. Concentrations and route of administration were chosen as described previously (8, 22).

### Measurement of Renal and Hematologic Function

Plasma urea was measured using an enzymatic method by the analyzer KONELAB (ThermoFisher Scientific). Microscopic hematuria was detected with Multistix® 8SG Urin Strips (Siemens) using the following scale: negative, trace, 1+, 2+, 3+, and 4+. Urine protein and urine creatinine were measured by KONELAB on urine samples, in order to calculate the urine protein to creatinine ratio (UP/CR), used as a glomerular dysfunction marker. Hematocrit is the ratio between plasma volume after centrifugation and fresh blood volume collected from the mice.

### Immunohistochemistry and PAS Coloration of Mouse Tissues

Four-μm-thick paraffin-embedded sections were cut with a microtome (RM2235, Leica Biosystem). Antigen retrieval was performed in a low pH buffer using the PT-Link™ Dako (Agilent Technologies). VCAM-1 (Abcam, ab134047) was stained and revealed with a one-step polymer goat anti-rabbit IgG coupled with horseradish peroxidase (HRP) (Dako Envision™, Agilent Technologies, #K4003). Staining was visualized using diaminobenzidine (DAB). Slides were scanned by Nanozoomer® (Hamamatsu) and analyzed with NDPview2® (Hamamatsu). Periodic acid–Schiff coloration was performed by routine procedures using sections of paraffin-embedded kidneys. Coloration of slides was scanned by Slide Scanner Axio Scan (Zeiss).

### Immunofluorescence of Mouse Tissue

Five-μm-thick frozen sections of kidneys were cut with Cryostat (Leica AS-LMD, Leica Biosystem,) and fixed in acetone on ice for 10 min. C3 (Hycult Biotech, HM1065), C5b-9 (Abcam, ab55811), polyclonal anti-serum to human FH (Quidel, A312), goat IgG (Jackson Immunoresearch, 005-000-003), C5aR1 (Hycult Biotech, mAb 20/70), CD31 (Abcam, ab124432), and NGAL polyclonal IgG (R&D system, AF1857) were stained for 2 h at RT and revealed by secondary Donkey anti-Goat AF647 (ThermoScientific, A-21447) or goat anti-rat AF647 (ThermoScientific, A-21434). Nucleus were stained with DAPI. Stained tissues were scanned by Slide Scanner Axio Scan™ (Zeiss) and analyzed with Zen lite™ software (Zeiss).

### Elisa for Detection of Human FH in Mouse Plasma

We used polyclonal antibody against FH (IgG purified in house from the goat anti-FH antiserum, Quidel A312), which was left native or was biotinylated. The native anti-FH antibodies was coated on a 96-well plate overnight at 4°C. After washing with PBS Tween 0.1% and blocking with PBS BSA 1% for 1 h at RT, the mouse plasma was added to the plate and incubated for 1 h at RT. After washing, plate was incubated with the biotinylated anti-FH antibody for 1 h at RT. After additional washings, streptavidin-HRP (Dako, P039701-2) was added for 1 h at RT. FH concentration was revealed by SureBlue TMB microwell peroxidase substrate (KPL) and the reaction was stopped by sulfuric acid 2 mol/L. Multiskan Ex (Thermofisher Scientific) was used to read the optical density at 450 nm. Results are expressed in μg/mL according to the standard curve, made using commercial purified FH (Comptech).

### Measurement and Detection of Mouse FH and C3

Plasma FH and C3 levels were evaluated by WB using the Invitrogen™Novex™ (ThermoFisher Scientific) separated on Bis-tris gels and transferred onto nitrocellulose membranes, using diluted plasma at 1/50. Next, the membranes were blocked and incubated at 4°C overnight with an anti–human FH antiserum (Quidel, A312), an anti–serum against mouse C3 (MP Biomedicals, 0855463). Staining was revealed with HRP-linked secondary rabbit anti-goat antibody (Thermofisher, **#**31402) and detected by chemiluminescence with myECLimager® (Thermoscientific).

### mRNA Level Analyses

Frozen kidney sections were recovered in RLT buffer (Macherey-nagel) + 1% β-mercaptoethanol (Gibco, Thermoscientific) and used for mRNA extraction using RNAse-free NucleoSpin® RNA (Macherey-nagel). The quality and quantity of mRNA were evaluated with the Agilent 2100 bioanalyzer using the Agilent TNA 6000 NanoKit (Agilent Technologies), followed by retrotranscription to cDNA (Qiagen).

Gene markers of acute tubular injury (NGAL and Kim-1), of endothelial activation (ICAM-1, VCAM-1, P-selectin, E-selectin), of inflammation (IL-6, Ki67, cNOS, Endothelin), of coagulation system (PAI-1), HO-1, and FH were analyzed by RT-qPCR (primers from ThermoFisher) using the Taqman 7900 (Life Technologies). Gene markers expression was analyzed with SDS 2.1® software (ThermoFisher), after normalization on actin housekeeping gene expression and comparison with gene expression from the pool of PBS-treated mice of each strain.

### Statistical Analyses

Results were analyzed using statistical software GraphPad Prism 5 using Mann–Whitney test, Two-way ANOVA or Kruskall Wallis tests as indicated in the figures legends. Statistical significance was defined as *p* < 0.05.

## Results

### Hemolysis Triggers Stronger Kidney Injury but With Delayed Onset in HepatoFH^–/–^ Mice

**Figure 1:**
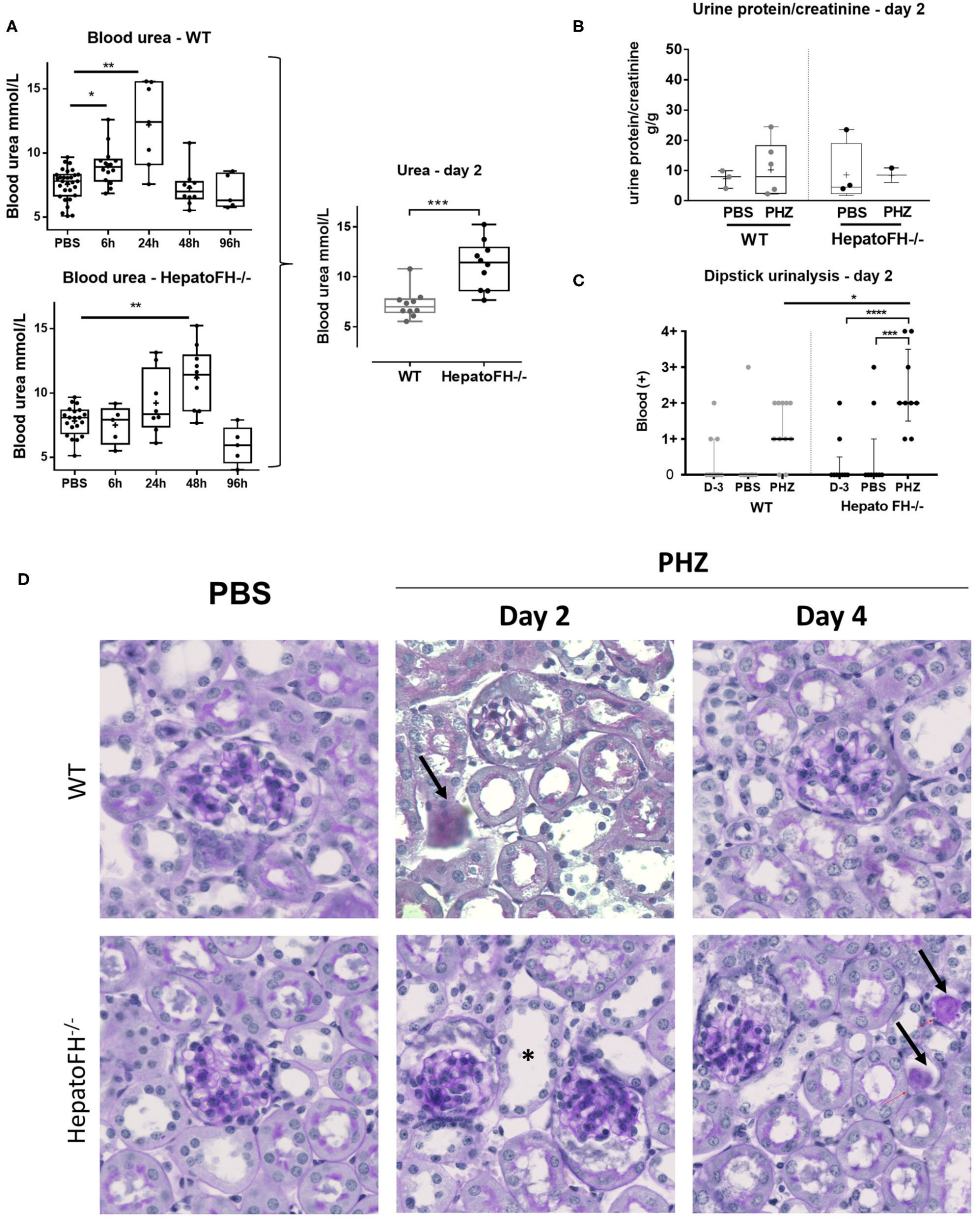
Hemolysis induces clinicopathologic changes in HepatoFH^−/−^ kidneys **(A–C)**. Evaluation of renal function by measurement of plasmatic urea level **(A)**, urine protein-creatinine ratio **(B)** and presence of blood in urine by urinary dipstick **(C)** in WT and hepatoFH^−/−^ mice, at 6, 24, 48, and 96 h after PBS or PHZ injection. The dipstick scale is in Figure S2. **(D)** PAS coloration of paraffin-embedded renal section (x40) after PBS or PHZ injection in WT (upper panel) hepatoFH^−/−^ (bottom panel) mice. Black arrows are showing the presence of casts inside the tubules. Asterix shows tubular injury with tubule dilation and loss of brush border. No significant changes are seen inside the glomeruli. *P*-values are derived from Two way ANOVA test with Sidak test correction: *p < 0.05, **p < 0.005, ***p < 0.001, ****p < 0.0001. The quantification values are box plots and dots with median and Min/Max points. Data from four independent experiments, each experimental group containing between 5 and 10 mice were pooled. PBS groups are composed of PBS-treated mice from each time points assessed with PHZ treatment. PBS-treated mouse were statistically equivalent from one another when analyzed over time. D, day.

Well-characterized model of PHZ-induced intravascular hemolysis (8, 23) was induced in mice with a hepatic deficiency of FH (hepatoFH^−/−^ mice) and in WT mice, described by Vernon et al. (21). Comparison between WT and hepatoFH^−/−^ mouse strains revealed similar levels of hematocrit at day 2 (Figure S1B), showing that lack of FH does not impact on hemolysis induction. Blood urea increased in WT mice at 6 h and day 1 post treatment and returned to normal at day 2 and 4. HepatoFH^−/−^ mice experienced delayed onset after PHZ treatment, with normal urea at 6 h, a trend toward increase at day 1, a peak at day 2, and normalization at day 4 (Figure 1A). Blood urea was higher at day 2 in hepatoFH^−/−^ compare to WT mice. Concomitantly, urine protein/creatinine ratio was not altered by PHZ treatment in both strains (Figure 1B). Dipstick urinalysis revealed no difference in WT mice between untreated, PBS- and PHZ-treated mice (Figures 1C, S2). However, a positive dipstick for blood was found in hepatoFH^−/−^ after PHZ treatment at day 2 compare to PBS and untreated hepatoFH^−/−^ mice, ^−^but also compared to WT mice after PHZ treatment (Figure 1C). There was no evidence of glomerular lesions, such as hypercellularity, double contours or mesangiolysis. However, there was a clear damage in the tubulointerstitial area in hepatoFH^−/−^ upon PHZ treatment, with a dilatation of tubular lumens and loss of brush border in proximal tubules at day 2 and tubular casts formation at day 4 (Figure 1D).

### Hemolysis Promotes Gene Expression of Acute Tubular Injury Markers in HepatoFH^–/–^

**Figure 2:**
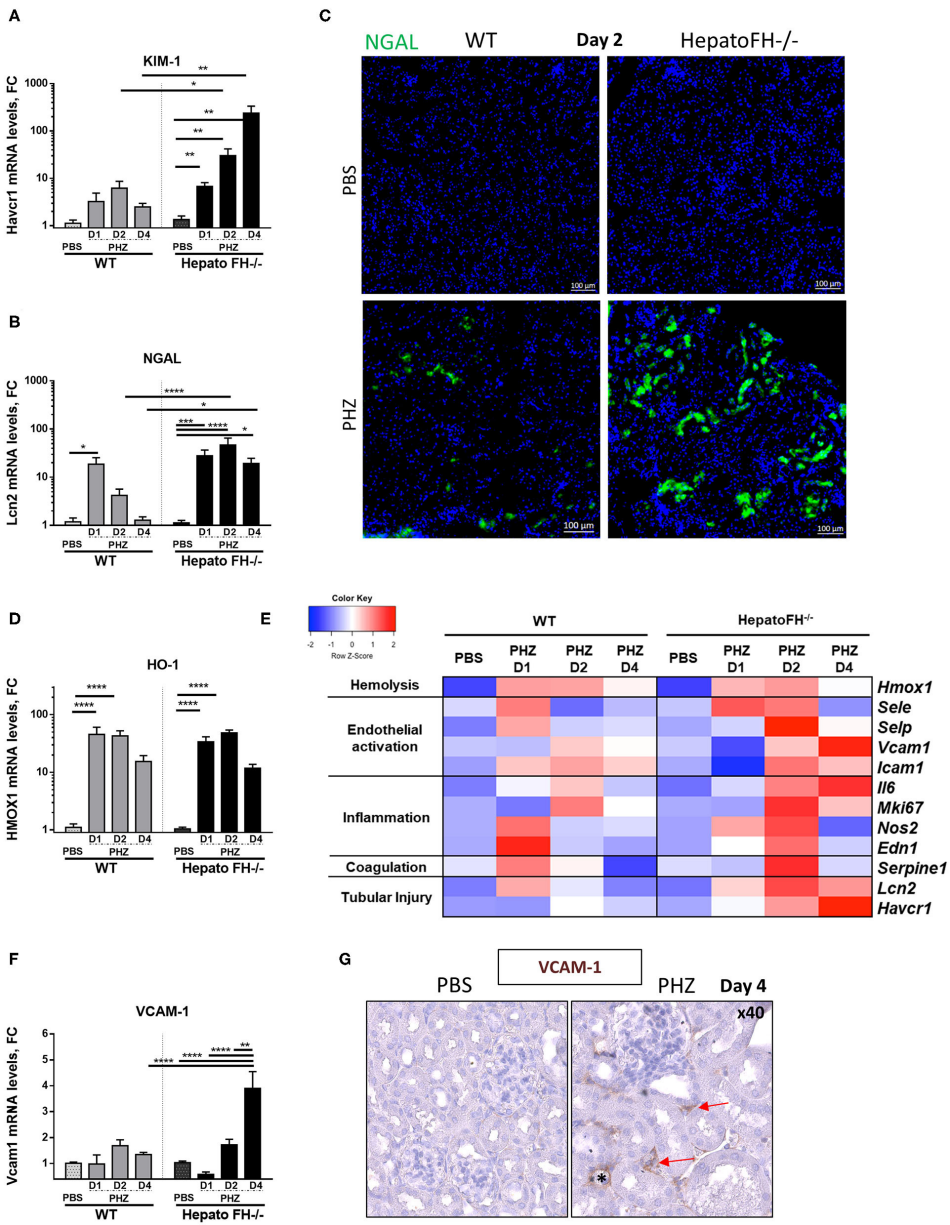
Factor H deficiency increases tubular injury after hemolysis. **(A,B)** Assessment of acute tubular injury by mRNA renal expression of Kim1 (*Havcr1*) **(A)** and NGAL (*Lcn2*) **(B)**, after PBS or PHZ injection (day 1, 2, and 4) in WT and hepatoFH^−/−^ mice. **(C)** Examples of NGAL staining (false green color) in tubular cells, by IF on frozen kidneys after PBS or PHZ injection in WT and HepatoFH^−/−^ mice (5x). **(D)** Renal expression of Heme oxygenase 1 (HO-1, *Hmox1*) mRNA after PBS or PHZ injection (day 1, 2, and 4) in WT and hepatoFH^−/−^ mice. **(E)** Heatmap summarizing the impact of hemolysis in renal mRNA expression: assessment of hemolysis, endothelial activation, inflammation, coagulation system, and acute tubular injury by mRNA renal gene expression at day 1, 2, and 4 after PBS or PHZ in WT and hepatoFH^−/−^ mice: Endothelial activation reflected by renal mRNA expression of E-selectin (*Esel*), P-selectin (*Psel*), ICAM-1 (*Icam1*); Inflammatory status and coagulation system reflected by renal mRNA expression of Endothelin (*Edn1*), cNOS (*Nos2*), Ki67 (*Mki67*), IL6 (*Il6*), and PAI-1 (*Serpine1*). Each case represents the mean of mRNA values from mice with the same genotype and the same treatment. In red, the conditions were genes displaying the highest expression, and in blue the lowest expression. The heatmap incorporates for comparison the data from panels **(A,B,D,F)**. **(F)** Renal expression of VCAM-1 (*Vcam1*) mRNA after PBS orPHZ injection (day 1, 2, and 4) in WT and hepatoFH^−/−^ mice. **(G)** Validation by VCAM-1 immunohistochemistry staining in embedded paraffin (false brown color), seen in peritubular capillaries (red arrow) and arteriols (*) in hepatoFH^−/−^ at day 4 post PHZ vs. PBS injection (40x). *P*-values are derived from Two way ANOVA test with Sidak correction for multiple comparisons: *p < 0.05; **p < 0.005; ***p < 0.001; ****p < 0.0001. mRNA values are represented in fold change (FC), relatively to the mean expression of PBS treated mice for each strain. Values are represented as mean +/− SEM. Data from two independent experiments, experimental groups containing between 4 and 11 mice.

Hemolysis induces upregulation of acute tubular injury markers (8, 23, 24). Here, mRNA study revealed increased expression of proximal tubules injury marker KIM-1 at day 1, which was maintained at day 2 and declined at day 4 in WT mice after PHZ treatment (Figure 2A). Interestingly, KIM-1 expression constantly increased from day 1 to day 4 in hepatoFH^−/−^ and was higher compared to its control PBS and to WT mice.

Further, tubular injury marker NGAL expression in WT mice acutely expressed (peak at day 1 and return to normal at day 4) as in (23) (Figure 2B). In hepatoFH^−/−^, NGAL expression was increased at day 1, and reached a plateau at day 2. Moreover, NGAL expression was stronger in kidneys from hepatoFH^−/−^ mice compared to the baseline and to WT mice, even at day 4. This different level of expression was confirmed by IF staining of NGAL, where NGAL in tubules from hepatoFH^−/−^ was stronger compared to WT mice after PHZ treatment (Figure 2C). As a control, the heme detoxifying enzyme heme oxygenase 1 (HO-1) was equally upregulated in WT and in hepatoFH^−/−^ mice (Figure 2D).

### Hemolysis Induces Gene Expression of EC Stress Markers in HepatoFH^−/−^

Endothelial cells get activated during hemolysis (8, 23). Here, we compared mRNA expression of endothelial stress markers in kidneys of WT and hepatoFH^−/−^ mice (Figure 2E). P-selectin, E-selectin and cNOS increased at day 1 in WT mice after PHZ injection, and returned to baseline at day 2. At day 2, each of these markers increased in hepatoFH^−/−^ after PHZ treatment compared to their PBS control and compared to WT mice treated with PHZ. Interestingly, expression of VCAM-1 showed a different kinetic Whereas VCAM-1 mRNA expression was found stable in WT mice under hemolysis after 4 days, its expression significantly increased in hepatoFH^−/−^ mice at the latest time point (day 4) compared to PBS control and to WT mice treated with PHZ (Figure 2F). Interestingly, VCAM-1 was mostly found in peritubular capillaries and arterioles within kidneys, with no evidence of VCAM-1 staining within glomeruli (Figure 2G).

### The Contribution of Hepatic FH Deficiency to Acute Tubular Necrosis Is Trigger-Dependent

**Figure 3:**
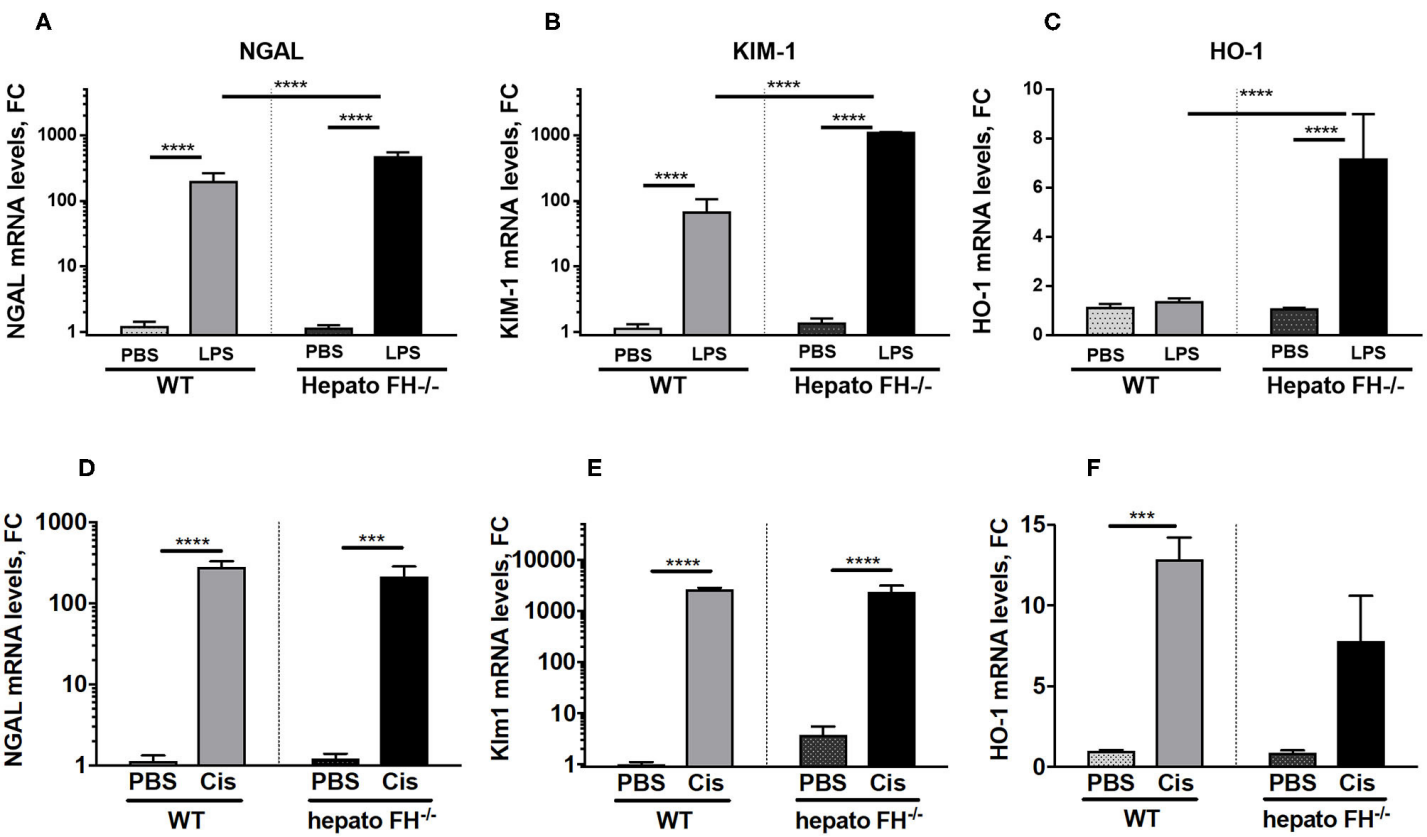
LPS-induced but not Cisplatin-induced tubular injury is exacerbated by FH deficiency. WT and hepatoFH^−/−^ received intraperitoneal injection of PBS, cisplatin (15 mg/kg) or LPS (LPS-EB from *E. coli* O111:B4) (2 mg/kg) at Day 0 and were sacrificed at Day 2. Renal mRNA expression in LPS treated mice of NGAL **(A)**, Kim-1 **(B)**, and HO-1 **(C)** or Cisplatin treated mice of NGAL (*Lcn2*) **(D)**, Kim-1 (*Havcr1*) **(E)** and HO-1 (*Hmox1*) **(F)** were measured by RTqPCR. *P-*values are derived from two way ANOVA with Sidak correction: ***P < 0.0005; ****P < 0.0001. Values are mean +/- SEM. Cis, cisplatin. Data from two independent experiments, experimental group containing between 2 and 5 mice.

Multiple agents induce AKI. To evaluate the impact of hepatic FH deficiency in other models of renal insult, we used models of LPS and cisplatin induced-nephrotoxicity (Figure 3). Both LPS (Figures 3A–C) and cisplatin (Figures 3D–F) triggered overexpression of NGAL, KIM-1 in WT mice at day 2. The expression was further enhanced in hepatoFH^−/−^ mice in the LPS model, while remained unaltered in the cisplatin one. HO-1 was upregulated by LPS in hepatoFH^−/−^ mice (Figure 3C) and in both strains by cisplatin (Figure 3F).

### Complement Activation Is Increased in Plasma and Within Glomeruli of HepatoFH^–/–^ Mice Upon Hemolytic Event

**Figure 4:**
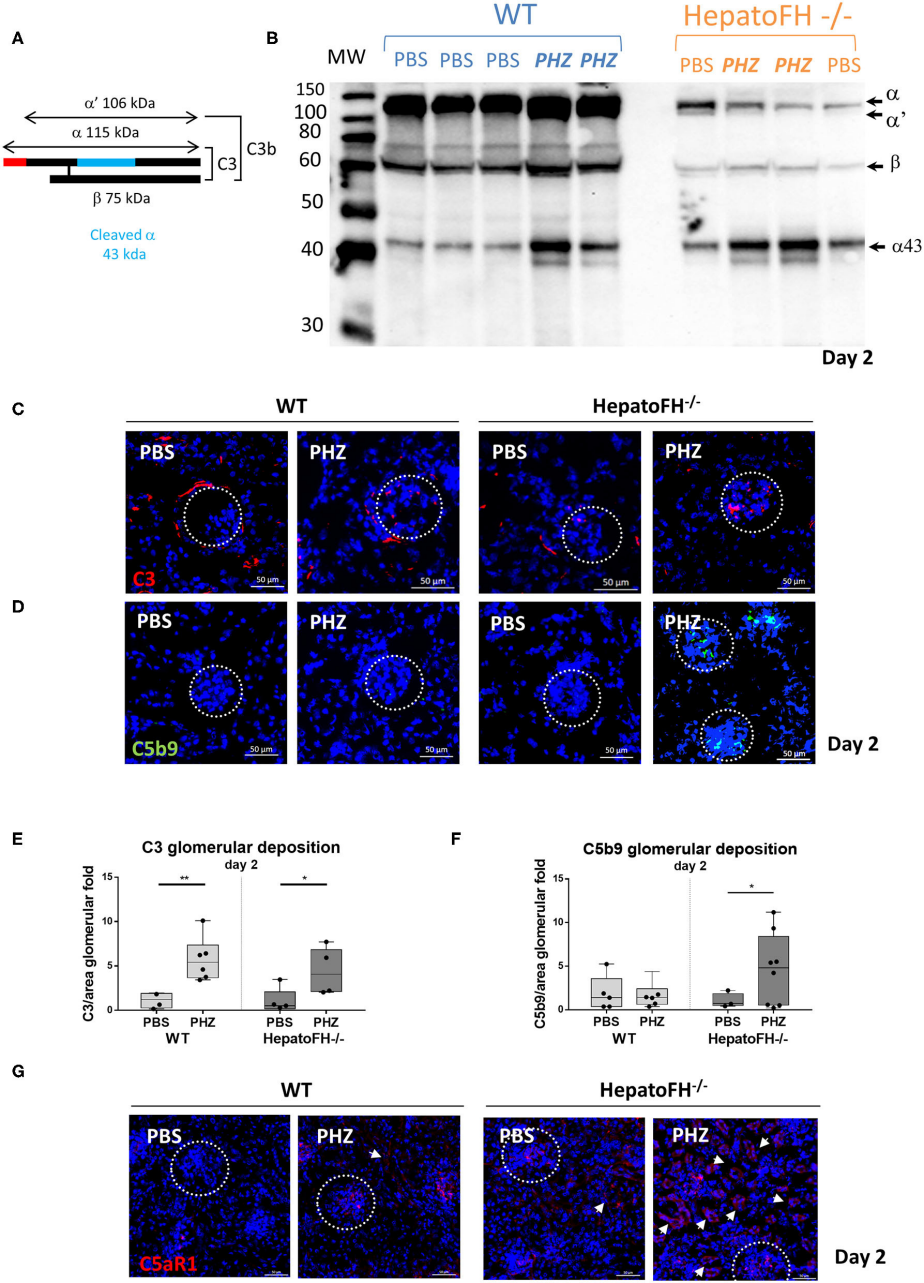
Lack of circulating FH leads to formation of the membrane attack complex in glomeruli and C5aR1 upregulation in tubules. **(A)** Schematic represetation of complement C3, indicating the different chains and cleavage fragments to be detected by WB. **(B)** Evaluation of C3 cleavage by WB after PBS or PHZ injection (Day 2) in WT and hepatoFH^−/−^ mice. Each lane correspond to 1 individual mouse. A mixture of purified C3 and C3b was used as a positive control to distinguish the presence of α and α' bands, characteristic for these two forms of C3, respectively. **(C,D)** Staining for C3 (false color red) **(C)**, and C5b-9 (false color green) **(D)** at day 2 after PBS or PHZ treatment in WT and hepatoFH^−/−^ mice. The glomeruli are indicated by a dotted white circle (x40). **(E,F)** Immunofluorescence quantification of glomerular C3 **(E)** and C5b-9 **(F)**. **(G)** Staining of C5aR1 (false color red) at day 2 after PBS or PHZ treatment in WT and hepatoFH^−/−^ mice. The arrowheads indicate the positive C5aR1 staining in the tubulointerstitium. In this figure, represetnative glomeruli are marked by a dotted circle. *P*-values are derived from Two way ANOVA test with Fisher test correction: *p < 0.05, **p < 0.005. The quantification values are represented in fold change (FC), relatively to the mean expression of PBS-treated mice for each strain. Values are box plots and dots with median and Min/Max points. D, day. Data from two independent experiments (except C5aR1, data from one experiment), experimental groups containing between 3 and 8 mice.

In agreement with Vernon et al. (21), the amount of plasma C3 in hepatoFH^−/−^ was strongly reduced compare to WT, as shown by WB (Figures 4A,B). Triggering hemolysis with PHZ resulted in plasma C3 consumption, as evident by the appearance of the α' and increase in the α43 band in the WT and in hepatoFH^−/−^ (day 2 data shown, Figures 4A,B). Further, we investigated the complement deposits in the kidneys (Figures 4C–F). Immunofluorescence staining of C3 revealed usual physiological traces of C3 fragment deposits surrounding the tubular cells in the WT mice, which disappeared in the hepatoFH^−/−^ mice due to the complement consumption (8, 21). No enhancement of the C3 fragments deposits were detected in the tubulointerstitium upon PHZ injection neither in the WT mice [in agreement with our previous studies (8, 25)] nor in the hepatoFH^−/−^ mice (data on day 2, Figure 4C). In contrast, the C3 fragments deposits were seen in glomeruli, which increased in both strains after PHZ treatment in a similar manner (Figures 4C,E). However, if C5b-9 was absent in kidneys in PBS-treated mice in WT and hepatoFH^−/−^ mice, and WT mice injected with PHZ, it was found elevated exclusively in glomeruli of hepatoFH^−/−^ after hemolysis at day 2 (Figures 4D,F). Even though we were not able to detect complement activation in tubular area with the detection limits of our methods, we found positive staining for C5aR1 on the WT mice, which was exacerbated in the tubules of the hepatoFH^−/−^ mice, injected with PHZ (Figure 4G). The PBS-injected WT mice showed limited/no staining and only in glomeruli. It increased after treatment with PHZ. The hepatoFH^−/−^ showed basal level of C5aR1 staining in the glomeruli and traces in the tubulointerstitium. The clear tubular staining appeared in these mice only after PHZ injection, making the tubular cells particularly responsive to C5a in this context.

### FH Is Present in the Mouse Kidney

**Figure 5:**
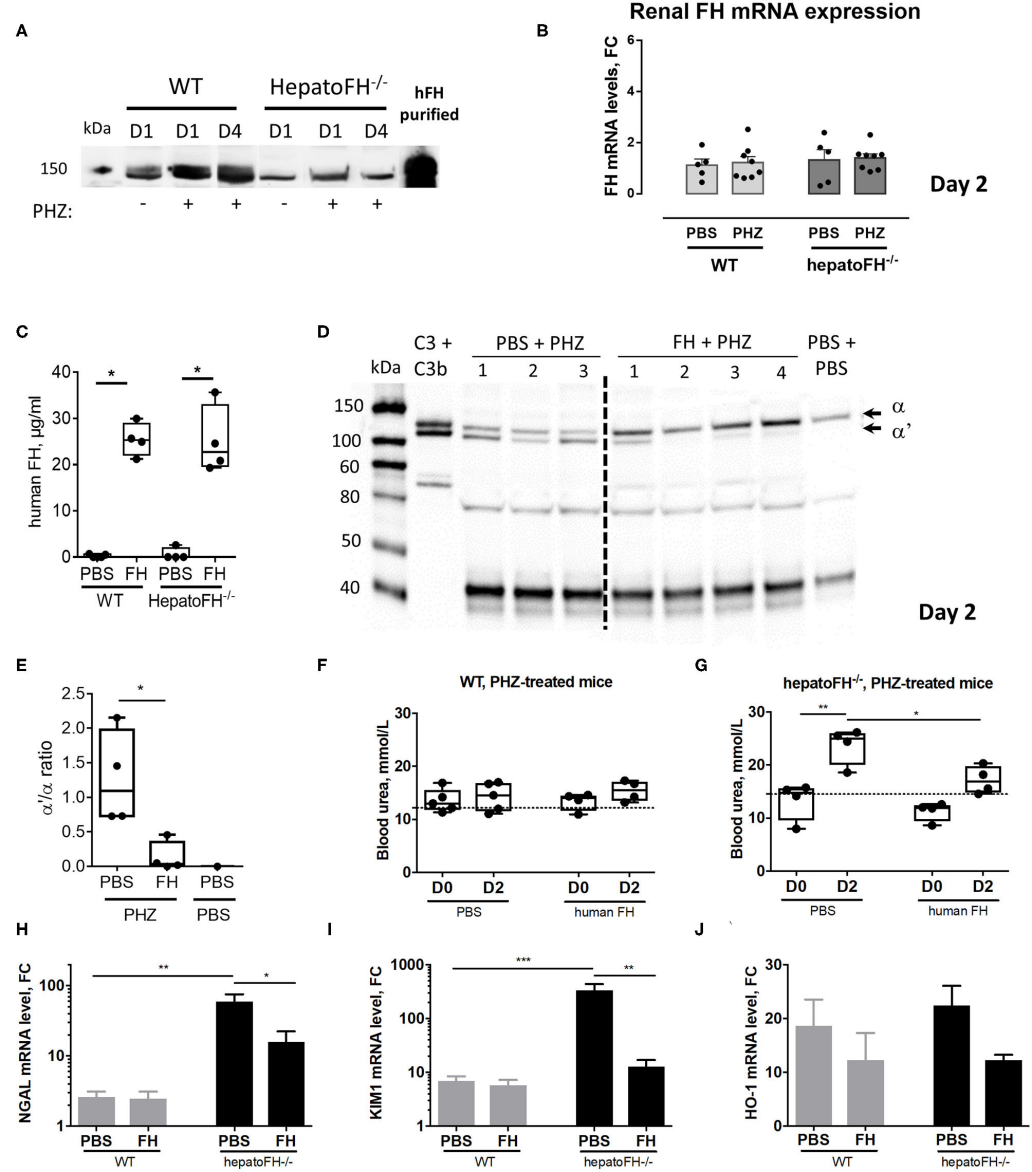
Purified FH controls kidney injury in hemolytic conditions. **(A)** Measurement of plasma FH by WB after PBS or PHZ injection (Day 1 or 4 post-injection) in WT and hepatoFH^−/−^ mice. Each lane correspond to 1 individual mouse. **(B)** Assessment of mRNA renal expression of FH in total kidney tissue. **(C)** Plasma human FH was assessed by ELISA at day 2 in WT and hepatoFH-/- mice, injected with human FH or PBS. **(D)** Plasma of hepatoFH^−/−^ mice was resolved by electrophoresis and probed for C3 cleavage to C3b in hepatoFH^−/−^ mice, treated or not with purified human FH. Each lane correspond to one individual mouse. **(E)** Quantification of the α'/α bands ratio, showing the absence of C3 activation in case of FH administration. **(F,G)** Blood urea was measured in plasma samples collected from PHZ-injected WT **(F)** or hepatoFH^−/−^ **(G)** mice, pre-treated with human FH or PBS, at day 0 (D0) and day 2 (D2). **(H–J)** Assessment of mRNA renal expression for NGAL (*Lcn2*) **(H)**, Kim-1 (*Havcr1*) **(I)** and HO-1 (*Hmox1*) **(J)** at day 2 in PHZ-treated WT and hepatoFH^−/−^ mice pre-treated with human FH or PBS. *P*-values are derived from Two way ANOVA test with Sidak correction for multiple comparisons: *p < 0.05; **p < 0.005; ***p < 0.001. Plasma parameters are box plots and dots with median and Min/Max points. mRNA values are represented in fold change (FC), relatively to the mean expression of PBS-treated mice for each strain. Values are represented as mean +/- SEM for the gene expression and SD for the remaining panels. Data obtained from one experiment, experimental groups containing 4 mice.

The circulating FH was lower in hepatoFH^−/−^ compare to WT at resting state. In both mouse backgrounds, an increase was observed after PHZ treatment (Figures 5A, S3A). Exploring the mRNA expression for *Cfh* in the kidney showed that the expression was identical in the WT and hepatoFH^−/−^ mice and was not affected by PHZ injection (Figure 5B). Exploring of FH staining in mouse kidney has been tricky and previous studies failed to detect it despite the clear evidence for mRNA production by various renal cell populations (26–31). The reason for this lack of staining in previous studies vs. the positive staining detected here is unclear and is likely related to the experimental conditions. Therefore, these staining results should be taken with caution, before being validated by an independent laboratory/method. Our conclusion that what we detect in the kidney is likely FH comes from the following arguments: Among the tested antibodies, we selected the anti-human factor H goat antiserum of Quidel, because it showed cross-reactivity with mouse FH by western blot (Figures 5A, S3A) with low non-specific binding to other proteins in plasma (Figure S3A). We tested its specificity for mouse FH in tissues, taking the advantage of the hepatoFH^−/−^ mice, which is invalidated for FH specifically in the hepatocytes (21). Upon optimization, the dilution 1/500 was selected, because it gave positive staining in the WT mice in hepatocytes and endothelial cells and positive staining in the EC of the hepatoFH^−/−^ mouse but not in its hepatocytes (Figure S3B). With more diluted antibody this signal was lost. Nevertheless, we cannot fully exclude that part of the staining could be attributed to mouse factor H related proteins, which are produced by the liver but not by the kidney, and could come from the circulation. The presumed FH staining of frozen kidney sections from WT mice revealed strong staining within the glomeruli and positive staining in the tubules, which was unaffected by the PHZ treatment (data from day 2, Figure S3C). The glomerular staining partly co-localized with EC marker CD31 (Figure S3D). The hepatoFH^−/−^ mice presented with a stronger staining in the glomeruli, likely due to binding of FH to the C3 fragments deposits present there at basal level, irrespective of the PHZ treatment. In contrast to the WT mice, the tubular staining in the hepatoFH^−/−^ mice was very weak, suggesting that at least in part, the FH on the tubular cells comes from the circulation (Figure S3C).

### Administration of Purified FH Prevents From Hemolysis-Driven AKI

To validate the importance of FH in the resistance to renal injury, we pre-treated hepatoFH^−/−^ mice with purified human FH, known to regulate mouse complement (22). We established that human FH was detectable in comparable plasma concentrations in WT and hepatoFH^−/−^ mice after a single intraperitoneal injection (Figure 5C). Furthermore, injected human FH was able to prevent from complement activation, as shown by WB on mouse plasma samples (Figure 5D) and bands quantification (Figure 5E). As a marker of AKI, we measured blood urea concentration 2 days after PHZ administration of WT and hepatoFH^−/−^ mice pre-injected with human FH or PBS as a control. Blood urea did not increase in WT mice after PHZ treatment at day 2 and was comparable to the PBS-treated mice (Figure 5F). Therefore, pre-treatment of those mice with human FH did not affect blood urea concentration. However, PHZ treatment induced an increase of blood urea in hepatoFH^−/−^ mice at day 2, which was prevented by pre-injection of human FH, normalizing to a similar level to PBS-treated mice (Figure 5G). Moreover, pre-injection of human FH partially prevented the increase of NGAL and KIM-1 expression in hepatoFH^−/−^ mice observed at day 2 after PHZ treatment. No effect was detected in WT mice, since these markers had already returned to near normal spontaneously (Figures 5H,I). Of note, pre-injection of FH did not affect significantly the increase of HO-1 expression in both WT and hepatoFH^−/−^ mice, suggesting that this marker is dependent on hemolytic status rather than complement-mediated tubular injury (Figure 5J).

## Discussion

Here we report the key role of systemic FH in protecting the kidneys against hemolysis-induced injury. Proximal tubules were particularly impacted by the reduced circulating FH and administration of purified FH partially prevented hemolysis-induced renal damage.

Despite the clear impact of intravascular hemolysis on the kidneys, hemolytic WT mice did not develop severe kidney failure (8, 23), suggesting an efficient underlying control mechanism. Since we have found that complement plays a major role in the hemolysis-induced kidney injury, we hypothesized that the tight regulation imposed by FH would contribute to renal protection. Interestingly, the disease course followed a different kinetic in the WT and hepatoFH^−/−^ mice, with hepatoFH^−/−^ mice having later onset but showing a more severe and persistent phenotype. The later onset could be explained by the partial C3 consumption, which was initially protective, since there was not enough C3 to be cleaved and deposited in order to promote the terminal pathway. Indeed, we found that C3^−/−^ mice had attenuated expression of genes, triggered by intravascular hemolysis (8) and FH deficient mice were partially protected from ischemia/reperfusion injury due to the secondary consumption and therefore deficiency of C3 (19).

Hemolysis leads to pronounced tubular injury (2, 32–35). Complement AP activation is an important pathogenic mechanism in the development of AKI, especially in the tubulointerstitium, where it has been shown to drive severe injury (19, 20, 36). Moreover, we have recently demonstrated that in hemolytic conditions tubular injury markers were downregulated in C3^−/−^ mice, confirming the harmful effect of complement activation on tubular cells (8). In line with these findings, we here detected further increases of NGAL and KIM-1 in hepatoFH^−/−^, which were maintained at high levels at day 4 compared to WT mice. Furthermore, VCAM-1 was upregulated in peritubular capillaries in hepatoFH^−/−^ mice at day 4.

The role of complement for the tubular injury during intravascular hemolysis may seem counterintuitive, since the deposition of complement activation fragments C3b/iC3b, as well as C5b-9 in the hepatoFH^−/−^ mice, occurs in the glomeruli and not on the tubules. Plasma FH protects the glomerular endothelium in the WT mice, since the cascade does not proceed to the terminal pathway during a hemolytic event, contrary to the hepatoFH^−/−^ mice. However, processing of C3 and assembly of C5b-9 within glomeruli strongly suggest local endothelium damage and release of the anaphylatoxins C3a and C5a with a respective molecular weight of 10 and 11kDa. Proteins of this molecular weight are physiologically freely filtered by glomeruli to urine chamber and reabsorbed by proximal tubules. Expression of C5aR1 on damaged tubular epithelium, as depicted here under hemolytic conditions, could be locally activated by the excessive amount of C5a, filtered through glomeruli, contributing to nephrotoxicity (37, 38). Systemic FH could thus reduce the formation of systemic C3a and C5a in the circulation, without reaching the interstitium. Another possibility is that FH is coming from the peritubular capillaries, reaching and protecting the tubules locally. This is supported by the decreased tubular staining for FH of the hepatoFH^−/−^ mice despite the identical intrarenal gene expression of *Cfh*. The intact endothelial barrier of peritubular capillaries, though, is likely to be an important limiting factor for access of complement proteins, including FH, to the tubular epithelium. The activation of plasma-derived complement at the basolateral border of tubules of mice in a model of ischemia/reperfusion injury, though, supports the idea of a diffusion of complement proteins from blood to the interstitium (39). Binding of the plasma-derived FH to the tubular cells could protect them from the hemolysis-mediated oxidative stress, by a mechanism, similar to the one described for the retinal pigment epithelial cells (40). This possibility requires further instigation. Nevertheless, our data shows that circulating FH is necessary to protect from acute tubular injury. This conclusion is supported also by the protective effect of the systemic injection of FH in the hepatoFH^−/−^ mice against overexpression of tubular injury markers as well as complement activation in the circulation.

Besides tubular injuries, the glomerular function was particularly difficult to analyze in our model since hemoglobinuria might be a confounding factor for both, proteinuria and hematuria. We found a slight increase of blood in urines of hepatoFH^−/−^ mice treated by PHZ in comparison to PHZ-treated WT mice, but without changes in light microscopy aspect. Thus, our histological results argue that tubular area is more susceptible to hemolysis related-damage than glomeruli, in agreement with previous studies (20, 41, 42). The glomerular cells, including the endothelial ones, produce FH, which likely contributes to the protection and prevents from exacerbated complement activation, even in case of reduced FH levels in plasma.

To find out if the observed protective effect of FH is restricted to the systemic hemolysis-associated AKI found here, and to the ischemia/reperfusion AKI (19, 20), we induced AKI in hepatoFH^−/−^ mice by LPS-mediated sepsis and by cisplatin. LPS activates the AP (43) and triggers TLR-4 in a way similar to heme (9, 44). Therefore, mice injected with LPS showed the same sensitivity in regard to dysregulation of the complement system with aggravated tubular injury in hepatoFH^−/−^. By contrast, while cisplatin-induced tubular injury occurs in a TLR-4-dependent manner (45), it does not activate complement (46) and the AKI here was thus FH-independent. These results account for a context-dependent protection provided by FH. FH is not implicated in all forms of acute tubular necrosis but likely restricted to complement triggering event, such as the ischemic tissue, LPS and heme.

Overall, these results demonstrate the importance of systemic FH for renal protection and raise the possibility of using purified FH or FH-derived molecules as therapeutic agents (22, 47–51) in hemolytic conditions. Indeed, pre-treatment of the hepatoFH^−/−^ mice with FH prevented over-activation of hemolysis-mediated complement and the upregulation of the tubular injury markers.

In summary, this study highlights the specific role of FH to protect against AKI in case of systemic intravascular hemolysis. Liver-produced FH seems to be a key protective factor for tubular cells. Based on our data, we propose the following model. During intravascular hemolysis the activation of the complement cascade occurs systemically and on glomerular endothelium. Liver-derived FH binds to the glomerular EC, injured by heme and other hemolysis-derived products, and prevents formation of the membrane attack complex C5b-9. C3a and C5a are, though, generated locally and filter through the glomerular filtration barrier to reach tubulointerstitium. Hemoglobin dimers and heme also reach tubulointerstitium, activating the tubular cells. The tubular cells overexpress C5aR1 and become responsive to the filtered C5a, aggravating thus the tissue injury. Improvement of our understanding on the kidney protection mediated by FH opens a door for the development of future potential complement therapy for tubular injury in kidney diseases either related to complement dysregulation or intravascular hemolysis.

## Data Availability Statement

The original contributions presented in the study are included in the article/Supplementary Material, further inquiries can be directed to the corresponding author/s.

## Ethics Statement

Experimental protocols were approved by Charles Darwin ethical committee (Paris, France) and by the French Ministry of Agriculture (Paris, France) number APAFIS# 3764-201601121739330v3. All the experiments were conducted in accordance with their recommendations for care and use of laboratory animals. Written informed consent was obtained from the owners for the participation of their animals in this study.

## Author Contributions

LR and NM designed the research. NM, JL, VP, AG, IB, SK, TR-R, and CT performed the research. MP provided HepatoFH^−/−^ mice. LR, NM, JL, AG, VP, and IB analyzed the data. LR, NM, JL, AG, VP, VF-B, SC, and MP discussed the data. LR, NM, JL, and AG wrote the manuscript. All authors contributed to the article and approved the submitted version.

## Conflict of Interest

The authors declare that the research was conducted in the absence of any commercial or financial relationships that could be construed as a potential conflict of interest.
