# Supplementary figures and images for: Circulating FH Protects Kidneys From Tubular Injury During Systemic Hemolysis
Source: Front Immunol. 2020 Aug 7;11:1772. doi: 10.3389/fimmu.2020.01772 (PMC7426730; doi:10.3389/fimmu.2020.01772)

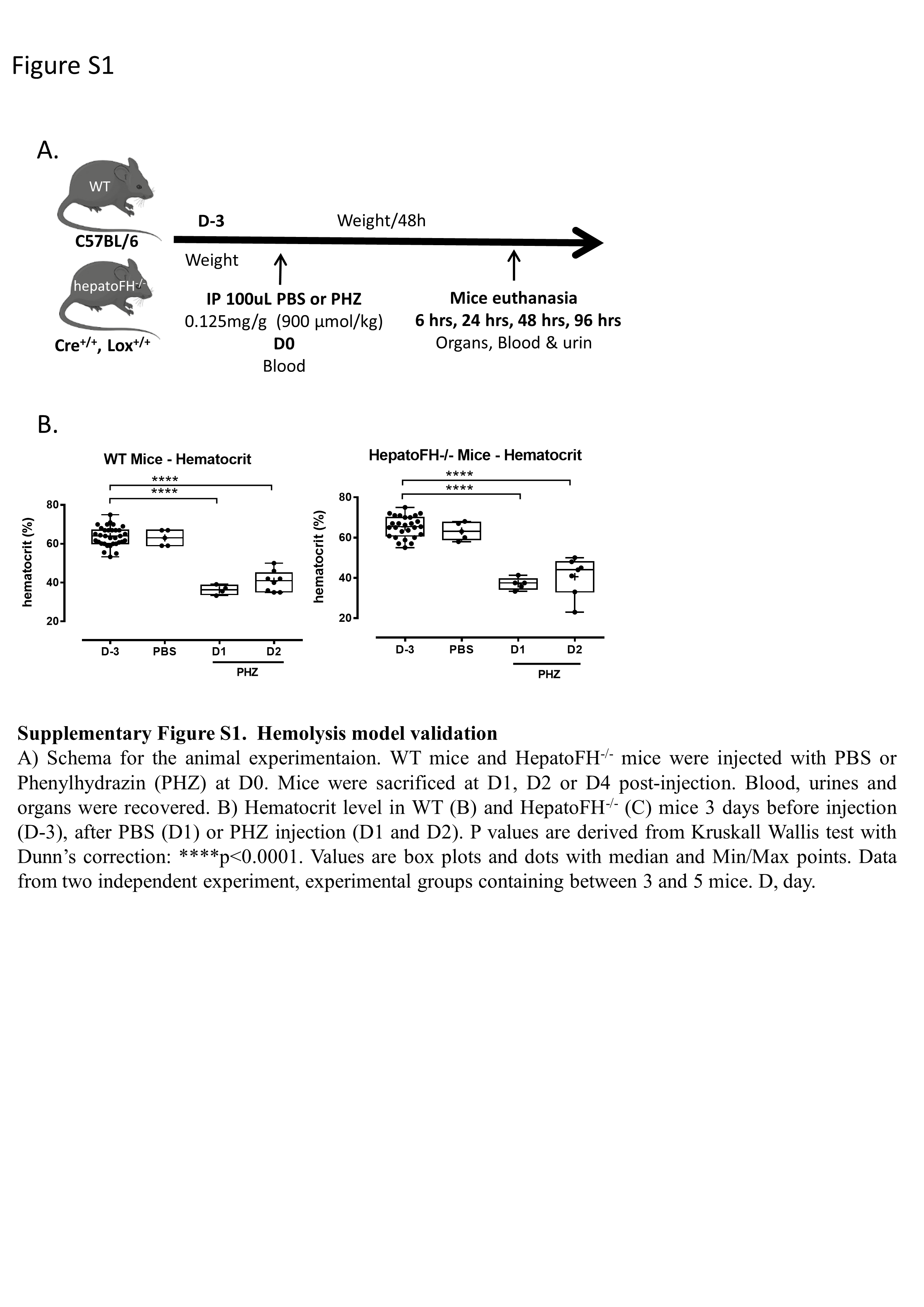

Supplement: Supplementary file 1 [file Image_1.TIF]

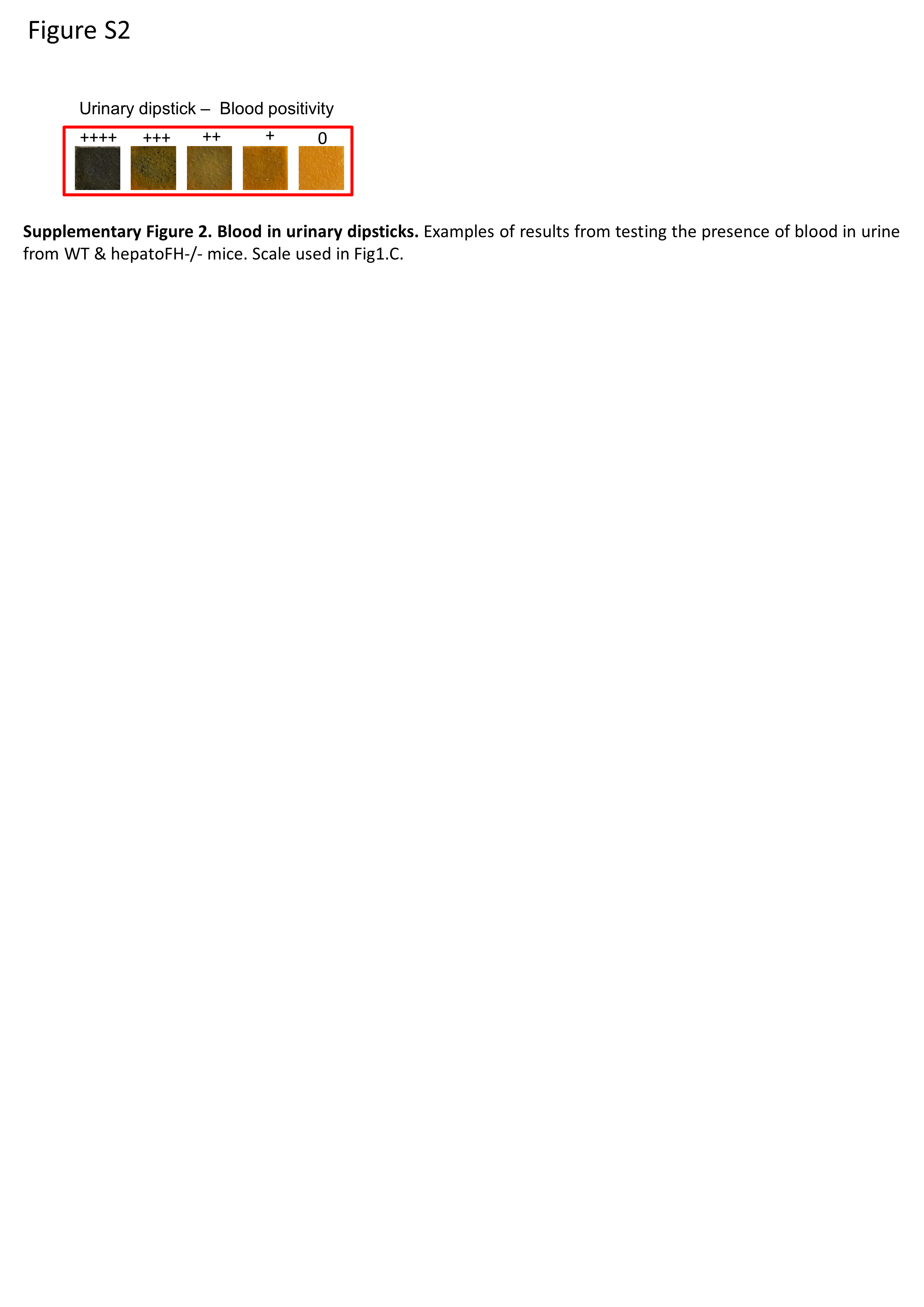

Supplement: Supplementary file 2 [file Image_2.TIF]

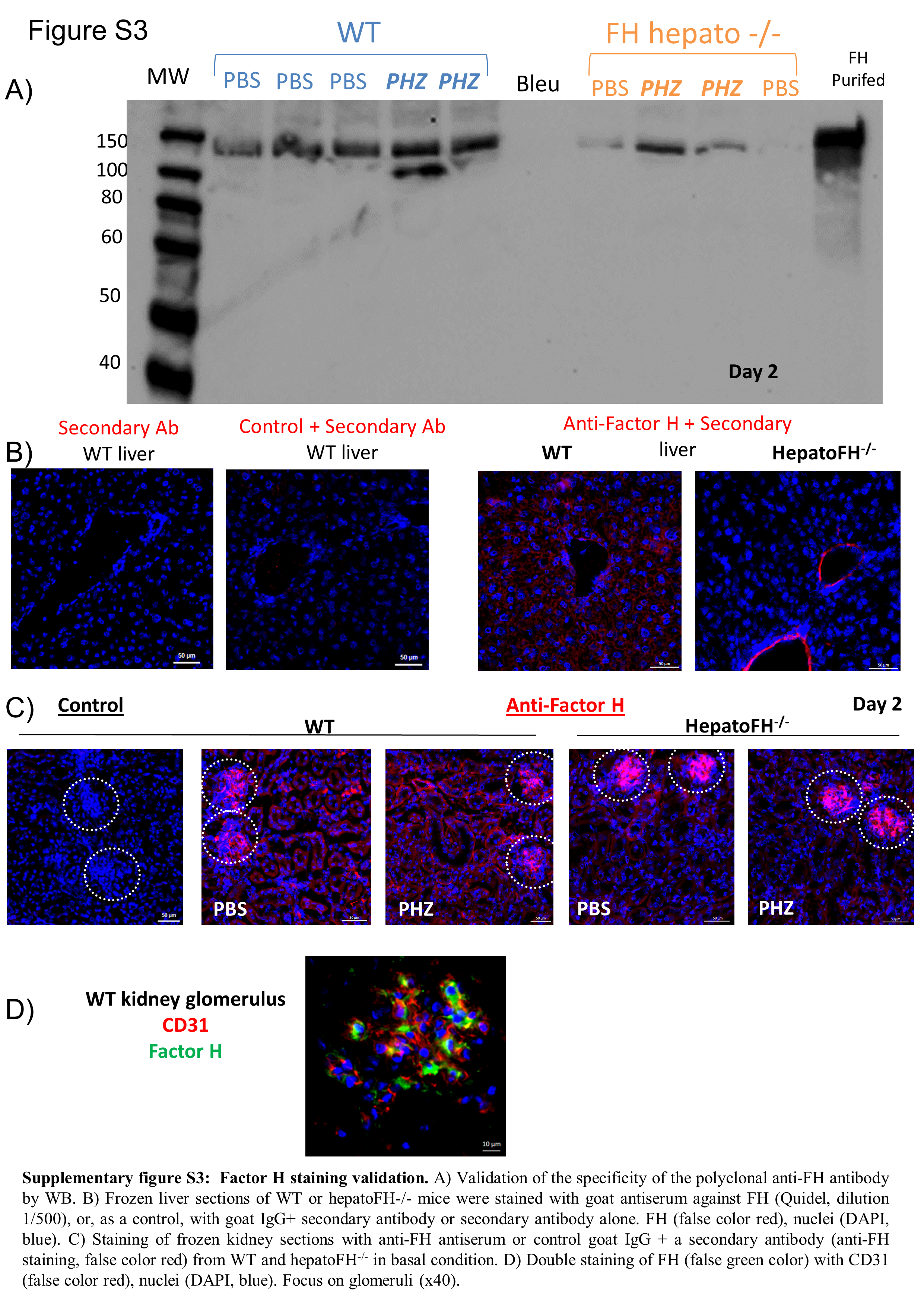

Supplement: Supplementary file 3 [file Image_3.TIF]
